# Odanacatib, a Cathepsin K Cysteine Protease Inhibitor, Kills Hookworm In Vivo

**DOI:** 10.3390/ph9030039

**Published:** 2016-07-04

**Authors:** Jon J. Vermeire, Brian M. Suzuki, Conor R. Caffrey

**Affiliations:** Center for Discovery and Innovation in Parasitic Diseases, Department of Pathology, University of California San Francisco, San Francisco, CA 94158, USA; jon.vermeire@gmail.com (J.J.V.); bmsuzuki@ucsd.edu (B.M.S.)

**Keywords:** parasite, hookworm, soil-transmitted helminth, cysteine protease, K11777, odanacatib, Merck, anthelmintic

## Abstract

Hookworm infection is chief among soil-transmitted helminthiases (STHs) for the chronic morbidly inflicted. Deworming via mass drug administration (MDA) programs most often employs single doses of benzimidazole drugs to which resistance is a constant threat. To discover new drugs, we employ a hamster model of hookworm infection with *Ancylostoma ceylanicum* and use albendazole (ABZ; 10 mg/kg orally) as the gold standard therapy. We previously showed that a single oral 100 mg/kg dose of the cathepsin cysteine protease (CP) inhibitor, K11777, offers near cure of infection that is associated with a 95% reduction in the parasite’s resident CP activity. We confirm these findings here and demonstrate that odanacatib (ODN), Merck’s cathepsin K inhibitor and post-clinical Phase III drug candidate for treatment of osteoporosis, decreases worm burden by 73% at the same dose with a 51% reduction in the parasite’s CP activity. Unlike K11777, ODN is a modest inhibitor of both mammalian cathepsin B and the predominant cathepsin B-like activity measureable in hookworm extracts. ODN’s somewhat unexpected efficacy, therefore, may be due to its excellent pharmacokinetic (PK) profile which allows for sustained plasma exposure and, possibly, sufficient perturbation of hookworm cathepsin B activity to be detrimental to survival. Accordingly, identifying a CP inhibitor(s) that combines the inhibition potency of K11777 and the PK attributes of ODN could lead to a drug that is effective at a lower dose. Achieving this would potentially provide an alternative or back-up to the current anti-hookworm drug, albendazole.

## 1. Introduction

Soil-transmitted helminthiases (STHs) caused by parasitic nematodes are associated with extreme poverty. Of these, hookworm disease afflicts as much as 7% of the world’s population, principally in sub-Saharan Africa, South America, and South and South-East Asia [1,2,3,4]. The disease is primarily due to infection by *Ancylostoma duodenale* or *Necator americanus*, and is manifested particularly in the under-nourished, causing or exacerbating iron-deficient anemia that can slow childhood physical development and cognition [5,6,7]. In addition, infection can slow fetal growth, and contribute to premature birth and maternal mortality [8,9,10]. Treatment and control of STHs employ periodic de-worming with drugs. The benzimidazoles, albendazole (ABZ), mebendazole, are most often used [11,12,13,14,15]. ABZ is the more effective at a single oral dose [16,17,18,19,20], making it suitable for mass drug administration campaigns [12,21,22]. However, concerns over drug resistance remain, particularly with the recent trans-national efforts to improve access to essential drugs [23,24]. Such unease is compounded by reports of less-than-anticipated cure rates with ABZ [25,26] (also reviewed in [27,28]), which in one case, was neither due to poor drug quality nor issues regarding patient compliance [25]. Thus, the need for new drugs (e.g., [29,30]) remains. 

**Figure 1:**
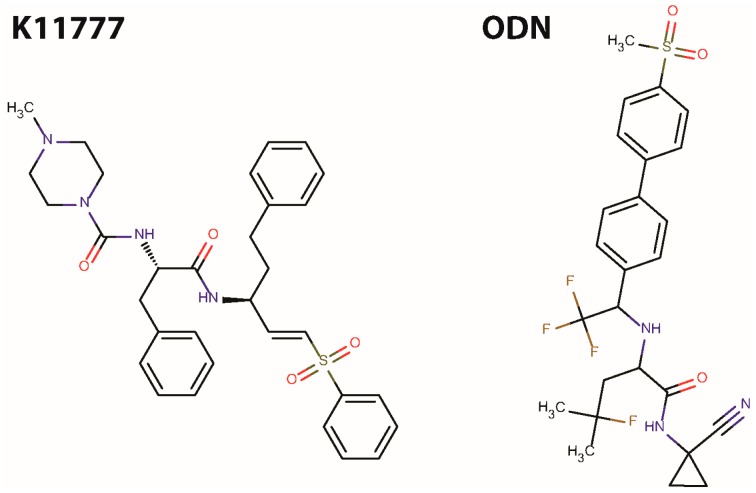
Structures of K11777 and ODN.

As a potential new class of anthelmintic, we are investigating small molecule protease inhibitors, specifically those that target cysteine-class cathepsins that are important to the survival of many parasitic organisms [31,32], including flatworms and nematodes [33,34,35,36]. We employ the Golden Syrian hamster infected with *Ancylostoma ceylanicum* to identify potential therapeutic agents: a single, oral 10 mg/kg dose of ABZ is our gold-standard drug regimen. Using this hamster model, we previously demonstrated that a single, oral 100 mg/kg dose of the cathepsin cysteine protease (CP) inhibitor, *N*-methyl-piperazine-phenylalanyl-homophenylalanyl-vinylsulfone-phenyl (K11777; Figure 1) [37], provided near-cure of hookworm infection [36]. In line with the inhibitor’s mechanism of action, the resident CP activity of worms harvested after treatment was decreased by 95% [36]. Although the data are encouraging, the effective dose is at least ten-times greater than that of ABZ. Accordingly, we continue to search for other CP inhibitors that either provide the necessary efficacy ‘as is’, i.e., without further chemical modification, or act as useful starting points for further development. Here, we describe the in vivo efficacy of Merck’s peptidomimetic, nitrile cathepsin K inhibitor, odanacatib (ODN; Figure 1), which has completed Phase III clinical trials for treatment of post-menopausal osteoporosis [38,39,40].

## 2. Results and Discussion

**Figure 2:**
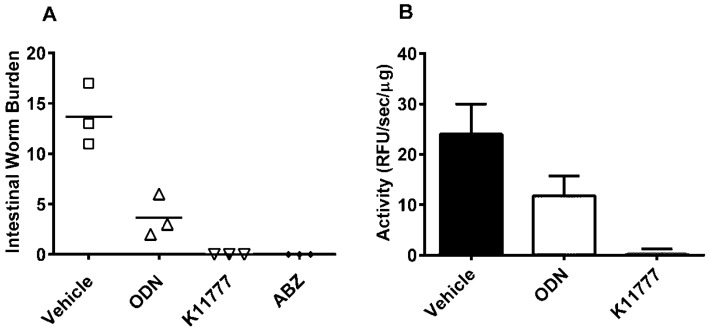
K11777 and ODN reduce *Ancylostoma* *ceylanicum* burdens in Golden Syrian hamsters and decrease the parasite’s resident CP activity. (**A**) Groups of hamsters (*n* = 3) were infected with 75 third stage *A. ceylanicum* larvae. At 18 days post-infection (DPI) hamsters were treated once orally with K11777 (100 mg/kg) dissolved in water, or with ODN (100 mg/kg) or ABZ (10 mg/kg) dissolved in PEG400. At 24 DPI, all hamsters were sacrificed and intestinal worms counted. Reductions in worm burdens by ODN and K11777 were statistically significant (one-way ANOVA: *p* < 0.05 and *p* < 0.01, respectively); (**B**) Hamsters (*n* = 1) were treated with single oral doses of K11777, ODN or the PEG400 vehicle as described in (**A**). Worms were harvested 8 h later and soluble extracts prepared. Specific cysteine protease activity (relative fluorescence units/min/mg soluble extract) was measured using the fluorogenic substrate Z-Phe-Arg-AMC. Data points are expressed as means ± S.D. values from a single experiment performed in triplicate.

A single, oral 100 mg/kg dose of the vinyl sulfone inhibitor, K11777, or 10 mg/kg of the current anti-hookworm drug, ABZ, cured *A. ceylanicum* infection in Golden Syrian hamsters (Figure 2A). The data are consistent with our previous findings [36]. The same 100 mg/kg dose of ODN decreased hookworm burden by 73% (Figure 2A). A standard assay for CP activity [36,41,42] using a dipeptidyl fluorogenic substrate was used to measure whether administration of K11777 or ODN 8 h prior to worm recovery decreased the parasite’s specific CP activity (i.e., activity as a function of protein concentration) relative to that measured after exposure to vehicle. For ODN and K11777, the worm protease activities were reduced by 51% and 96%, respectively (Figure 2B).

Based on the inhibition of the parasite’s CP activity, it seems that both inhibitors engage the hypothesized target, namely a group of gut-associated cathepsin B-like enzymes [43] which are the predominant protease activity measurable in hookworm extracts under the assay conditions employed [36]. The smaller reduction in specific protease activity in worms exposed in vivo to ODN (51% vs. 96% for K11777) is consistent with the inhibitor’s weaker, but still considerable, anti-parasite efficacy (73% vs. 100% for K11777). Indeed, ODN’s efficacy is surprising given that it is 4.5 orders of magnitude more potent against cathepsin K compared to cathepsin B (Table 1) with which the hookworm proteases, irrespective of infecting species, share greatest homology [36,43]. In contrast, K11777, as a non-specific inhibitor of CPs, has low nanomolar IC_50_ values against various mammalian cathepsins (Table 1; reviewed in [44]).

**Figure 3:**
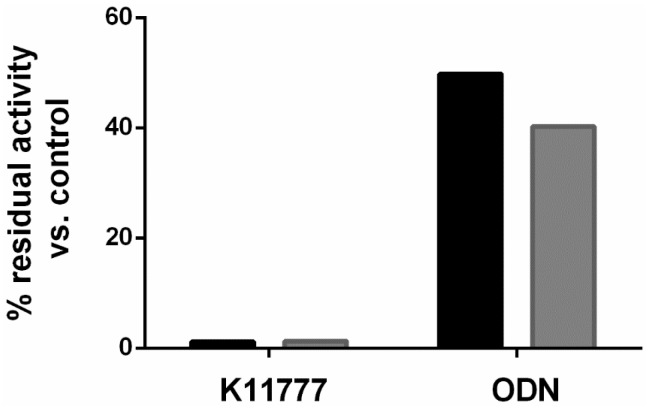
Inhibition of hookworm cysteine protease activity by K11777 and ODN. Soluble extracts of female (black bars) and male (grey bars) *A. ceylanicum* were incubated for 10 min with 1 μM inhibitor, as described in the text. Residual cysteine protease activity was measured with the fluorogenic substrate Z-Phe-Arg-AMC. Data were generated from two experiments each in duplicate; one experiment is shown.

Consistent with the poor inhibition of mammalian cathepsin B by ODN, we recorded modest 49.8% and 40.2% residual activities in soluble extracts of female and male hookworms, respectively, after a 10 min incubation with 1 μM ODN (Figure 3). In contrast, after incubation with K11777 at the same concentration, the respective hookworm cysteine protease activities were just 1.2% and 1.25% of the DMSO control. 

With its moderate inhibition of mammalian and hookworm cathepsins B, the question arises as to why ODN is as effective as it is in vivo. Part of the answer may lie in its outstanding pharmacokinetics (PK) profile which allows for once-weekly oral dosing of osteoporosis patients ([39,40]; Table 2); and which originally prompted us to test the inhibitor. The many attractive PK features of ODN include its low systemic clearance, long plasma half-life (T_1/2_) and good oral bioavailability (%F) in various pre-clinical animal models which conceivable would provide a sustained plasma loading to generate an anti-parasite effect (Table 2). In these same metrics, K11777, at a 20 to 50 times the dose in rats and dogs, respectively, was noticeably poorer. 

If PK is a key contributor to anti-hookworm efficacy, one might improve bioactivity by identifying small molecules that combine the nanomolar inhibition of the target cathepsin B proteases, as shown for K11777, with the attractive PK features of ODN. This idea could be initially explored by combining K11777 with ODN to identify potential synergistic or additive efficacy. Ideally, the combination of improved on-target potency and PK would result in significantly lowering the dose necessary to achieve cure, a vital goal, bearing in mind that the current drug standard, ABZ, is at least ten-fold more effective in the hamster model. Identifying a low dose CP therapy may offer an attractive chemical alternative or back-up to ABZ, in addition to the possibility of a combination therapy with ABZ. Finally, within the framework of designing an improved inhibitor, possible safety concerns regarding off-targeting of orthologous host proteases can also be addressed, mitigated by the knowledge that (i) treatment of hookworm infection will involve acute (single-dose) therapy only and (ii) the non-specific cathepsin inhibitor, K11777, continues to meet safety criteria as it progresses pre-clinically as a treatment for Chagas disease.

## 3. Experimental Section

### 3.1. Animals and Compounds

*A. ceylanicum* was maintained in male Golden Syrian hamsters (*Mesocricetus auratus*; Harlan Sprague Dawley, Somerville, NJ, USA) as described [27,47,48]. The animal protocol supporting this research was evaluated and approved by the University of California San Francisco’s Institutional Animal Care and Use Committee (IACUC) with the Approval number AN098756-02B. UCSF-IACUC derives its authority from the United States Public Health Service (PHS) Policy on Humane Care and Use of Laboratory Animals, and the Animal Welfare Act and Regulations (AWAR). All compounds were prepared immediately before administration to animals. ODN (MK-0822) was purchased from Chemietek (Indianapolis, IN, USA; CT-CG001) and ABZ from Sigma Aldrich (St. Louis, MO, USA; A4673). Prior to animal experiments ODN and ABZ were dissolved in 100% PEG400 whereas K11777-HCl was dissolved in deionized water [33,36].

### 3.2. Treatment Regimens and Cysteine Protease Activity Assay

Groups of hamsters (*n* = 4) were infected with 75 third stage *A. ceylanicum* larvae by oral gavage in 200 μL deionized water. Treatment regimens commenced at 18 days post-infection (DPI) to target adult worms [36] using 100 mg/kg ODN or K11777, or 10 mg/kg ABZ delivered by oral gavage in 200 μL vehicle. An infected vehicle group controlling for the PEG400 vehicle was also set up. On day 24 DPI, hamsters were sacrificed and their intestinal worm burdens counted [36].

To measure the effect of compounds on worm CP activity in vivo [36], one hamster from each of the treatment and vehicle groups was sacrificed 8 h post-treatment. Worms recovered were washed three times in RPMI 1640 and frozen at −80 °C prior to assay. Worms were thawed in 100 μL assay buffer (0.05 M sodium acetate, pH 5.5) and homogenized using RNase-free disposable pellet pestles and microtubes (Thermo Fisher Scientific, Waltham, MA, USA) for 10 min at room temperature. Homogenates were centrifuged at 5000 *g* for 10 min and the supernatants removed for analysis. Supernatants (1–2.5 μL) were mixed with 100 μL assay buffer containing 2 mM DTT in a black 96-well microtiter plate and left to stand at room temperature for 10 min. Then, 100 μL of assay buffer containing 2 mM DTT and 20 μM of the dipeptidyl fluorogenic substrate, benzyloxy carbonyl-phenylalanyl-arginyl-7-amido-4-methylcoumarin (Z-Phe-Arg-AMC) [41] was added with mixing. Linear rates of hydrolysis were followed in a FlexStation II (Molecular Devices, Sunnyvale, CA, USA) for 10 min. Protein concentrations of supernatants were measured using the micro-Bradford assay (BioRad, Hercules, CA, USA).

To measure the inhibition of hookworm CP activity in vitro, the assay was prepared as described above. K11777 and ODN (1 μL in DMSO to deliver 1 μM) were pre-incubated for 10 min with soluble extracts of either male or female *A. ceylanicum* worms (harvested 24 DPI) in a volume of 100 μL assay buffer prior to the addition of 100 μL of substrate solution.

## 4. Conclusions

The present data confirm the anti-hookworm efficacy of the CP inhibitor, K11777, in a small animal model of hookworm infection and extend the finding to include a structurally unrelated CP inhibitor, ODN. Based on differences in the CP inhibition and PK profiles between the two inhibitors, we reason that combining the CP inhibition potency of K11777 with the PK stability of ODN should lead to a CP inhibitor that is more effective at a lower dose thereby offering a possible drug alternative to or back-up for ABZ.

## Figures and Tables

**Table 1:** Inhibition of mammalian cysteine cathepsins and cruzain by CP inhibitors.

Inhibitor	Target Cathepsin and IC_50_ Value (nM)					
	CatB	CatF	CatK	CatL	CatS	Cruzain
K11777	9	3	1.8	<0.2	<0.2	3.5
ODN	1034	n.t.	0.2	2995	60	n.t.

Data for K11777 from [45] and for ODN from [38] as cited in [45]. Each assay was performed twice; n.t. = not tested; Cruzain is a cathepsin l-like protease in *Trypanosoma cruzi*, the etiological agent of Chagas disease [46].

**Table 2:** PK parameters for ODN and K11777.

Compound	Vehicle	Dose	C_max_	T_max_	AUC_0–∞_	T_1/2_	F
		(mg/kg)	(μM)	(h)	(μM·h)	(h)	%
ODN (rat)	100% PEG400	5	2.2 ± 0.4	1.8 ± 1.5	36 ± 10	5.8 ± 0.8	43 ± 12
ODN (dog)	60% PEG400	1	3.6	8	318	64	122
ODN (monkey)	Imwitor-Tween 80 (1:1)	5	0.3 ± 0.1	6 ± 2.3	4.8 ± 1.8	18 ± 4.3	18 ± 3.8
ODN (man)	capsule	25 ^1^	0.24 ± 0.052	14.2 ± 8.1	19.9 ± 4.1	96.7 ± 18.3	34
K11777 (mouse)	water	92	2.6	0.3	3.9	0.8	n.d.
K11777 (rat)	water	100	3.1	4	10.5	1.9	22
K11777 (dog)	water	50	1.4	0.34	1.0	0.5	15

Data for ODN are taken from [39,40] and represent means and SD values for the rat (*n* = 4), dog (*n* = 2), monkey (*n* = 4) and man (*n* = 6). In every case, a single oral dose was administered. Data for K11777 are from an internal pre-IND report from SRI International (2009). Data presented are means for the mouse (*n* = 3), male rat (*n* = 3) and dog (*n* = 2); n.d. = not determined; ^1^ Total dose (mg) administered.
